# Creating 'This is It' Moments in Gerontological Education

**DOI:** 10.1093/geroni/igab046.1053

**Published:** 2021-12-17

**Authors:** Candace Brown

**Affiliations:** Duke University, Durham, North Carolina, United States

## Abstract

We all have had “this is it” moments. And while many gerontologists will say they "fell" into the field, I solicit that educational paths are set before us and we have choice to which path that we take. Paths provide opportunities for discoveries about ourselves and in the case of gerontology, we have a breadth of choices. This is how I view teaching and why developing new courses in the field are a necessity. Change is constant; creating new paths for opportunities is optional. This lecture will provide how, as an educator, I accept my 'this is it' moments so I can effectively foster students to recognize their own 'this is it' moment(s) within gerontological education.

